# Osteoimmunomodulatory Effects of Zirconia‐Modified Titanium: Promoting Macrophage Activation and Osteoblast Mineralization at the Dental Implant Interface

**DOI:** 10.1002/cre2.70414

**Published:** 2026-07-19

**Authors:** Ottavia Cannatella, Biagio Matera, Francesca Rossi, Giovanni Passeri, Simone Lumetti, Ludovica Parisi, Benedetta Ghezzi

**Affiliations:** ^1^ Center of Dental Medicine University of Parma Parma Italy; ^2^ Department of Medicine and Surgery University of Parma Parma Italy; ^3^ IMEM‐CNR, Institute of Materials for Electronics and Magnetism‐National Research Council Parma Italy; ^4^ Laboratory for Oral Molecular Biology, Department of Orthodontics and Dentofacial Orthopedics University of Bern Bern Switzerland

**Keywords:** dental implants, inflammation, macrophages, osseointegration, osteoimmunomodulation

## Abstract

**Objective:**

Immune cells are the first to interact with implant surfaces, yet their contribution to osseointegration is often underestimated. Understanding how titanium‐based and zirconia‐enriched surface properties regulate macrophage polarization is essential for designing regenerative biomaterials. This study evaluates how these physicochemical characteristics modulate macrophage behavior and how macrophage‐derived signals affect osteoblast mineralization, a key determinant of successful clinical outcomes.

**Material and Methods:**

Human monocytes were differentiated into pro‐inflammatory (M1) or anti‐inflammatory (M2) macrophages on hydrophobic and hydrophilic titanium (P, SLA/modSLA) or zirconia‐enriched titanium (R/modR) surfaces. Macrophage polarization was assessed by Real Time‐PCR, fluorescent immunoassays, and Luminex analysis of pro‐/anti‐inflammatory cytokines and bone‐related markers. Conditioned media from M1/M2 macrophages cultured on each surface were used to culture human osteoblast‐like cells, and their differentiation and mineralization were evaluated by gene expression analysis and Alizarin Red staining.

**Results:**

The obtained results indicate that surface chemical composition, rather than hydrophilicity, is the main driver of early macrophage phenotype modulation. Zirconia incorporation induced effects comparable to titanium on macrophage activation while increasing transcription of pro‐ and anti‐inflammatory genes. Moreover, zirconia enhanced M1 activation and the release of angiogenic and osteogenic mediators, including VEGF and OPN. Although seemingly contradictory, these effects contribute to an immune microenvironment favorable to bone formation by promoting a balanced macrophage response. Consistently, zirconia‐enriched surfaces enhanced osteoblastic differentiation and mineralization, regardless of hydrophilic differences compared with conventional titanium surfaces.

**Conclusion:**

Our findings indicate that zirconia‐supplemented titanium alloys may provide a useful model to elucidate the concept of osseointegration as an osteoimmune process rather than a conventional bone healing response. The data emphasize the critical role of early M1 macrophage activity controlling the release of specific cytokines, supporting the hypothesis that the use of titanium/zirconia alloys may provide an osteoiummunomodulatory advantage that may contrast peri‐implant bone loss.

## 1 Introduction

Titanium has long represented the clinical gold standard for endosseous implants in dental and orthopedic applications. Yet, concerns related to its dark color have encouraged the development of zirconia‐enriched, whiter alloys. Zirconia‐based materials are well tolerated by surrounding tissues, display high chemical stability, offer higher compressive strength than titanium, and tend to limit bacterial adhesion and biofilm formation, thereby decreasing the risk of inflammatory reactions in peri‐implant tissues (Yin et al. 2017; Al‐Radha et al. 2012; Schünemann et al. 2019).

The clinical success of dental implants relies on a coordinated series of biological and mechanical events leading to the intimate integration of the implant with surrounding bone, known as osseointegration, a process that begins immediately after placement and whose progression is strongly influenced by surface properties (i.e., nano‐/microtopography and wettability) (Hotchkiss et al. 2016). In vitro basic research and clinical studies have confirmed that microrough, hydrophilic surfaces promote osteoblast differentiation and mineralization and accelerate osseointegration, improving implant stability when compared to smoother, less hydrophilic surfaces (Hotchkiss et al. 2016; Boyan et al. 2017; Parisi et al. 2022).

Historically, this evidence led to the assumption that bone‐forming cells were the primary drivers of osseointegration. However, recent pre‐clinical findings reveal that immune cells, particularly macrophages, are the first to interact with implant surfaces during early healing, initially dominating the tissue‐biomaterial interface, being replaced by osteoblasts only in a later phase, suggesting a previously underestimated crosstalk between immune and bone‐forming cells (Alvarez et al. 2016; Parisi et al. 2020). This shift calls for a re‐evaluation of the mechanisms underlying successful implant integration and recalls the concept of osteoimmune regulation.

Macrophages, key components of the innate immune system, are characterized by high plasticity that enables them to acquire either a pro‐inflammatory, antimicrobial phenotype (M1) or a pro‐regenerative, anti‐inflammatory phenotype (M2), depending on local cues (Lazarov et al. 2023; Chiu and Bharat 2016; Lee et al. 2013; Chen et al. 2023; Saqib et al. 2018).

Two main hypotheses have been proposed regarding macrophage behavior at implant interfaces: (i) implant surface features may induce stable, long‐lasting macrophage phenotypes; or (ii) surface characteristics may orchestrate a sequential recruitment of polarized macrophages, initially through M1 to resolve early inflammation, followed by M2 to promote tissue repair (Alvarez et al. 2016; Strizova et al. 2023). Both views highlight essential contributions of each phenotype during osseointegration. In vitro evidence suggests that surface roughness tends to favor M1 polarization, whereas hydrophilicity promotes M2. Still, the downstream consequences of macrophage polarization on osteoblast function and new bone deposition remain unclear.

Based on this background, the present study aimed to evaluate how implant surface characteristics influence macrophage polarization and how the resulting macrophage‐derived microenvironment affects osteoblast activation and bone formation.

## 2 Materials and Methods

### 2.1 Samples

Titanium or titanium/zirconia alloy discs of 15 mm in diameter and 1 mm in thickness were provided by Institute Straumann AG (Basel, Switzerland). Five different surfaces were used for this study, as detailed in Table 1. All experimental samples are standardized, commercially available medical‐grade materials, and their chemical composition, including the specific 85% Titanium/15% Zirconia ratio (Roxolid material), is certified and guaranteed by the manufacturer.

**Table 1:** Characteristics of the titanium and titanium/zirconia surfaces used in the study, classified according to chemical composition, surface treatment, and hydrophilicity.

Disc composition	Groups
Polished titanium surface	P
Sand‐blasted and acid‐etched titanium surface (SLA)	SLA
Sand‐blasted and acid‐etched hydrophilic titanium surface (SLActive)	modSLA
Sand‐blasted and acid etched 85% titanium and 15% zirconia surface (Roxolid)	R
Sand‐blasted and acid etched 85% titanium and 15% zirconia hydrophilic surface (Roxolid SLActive)	modR

### 2.2 Surface Hydrophilicity and Roughness

The wettability of the P, SLA, modSLA, R, and modR surfaces was evaluated by measuring the contact angles formed with a 10 μL water droplet deposited on the discs. Each measurement was performed three times, and the images were acquired the same number of times to ensure enough data points for statistical analysis. Contact angles were determined from standardized photographs using ImageJ (ImageJ, U.S. National Institutes of Health, Bethesda, MD, USA).

Surface roughness was measured using a contact profilometer (Tencor Instruments) and evaluated with the Ra parameter (Figure S2). This method involves a diamond stylus that moves both vertically and laterally across the sample surface. As it traverses the sample, the stylus’ height generates an analog signal, which is then digitized, processed, and displayed for final analysis.

### 2.3 Cell Culture

Human monocytes THP‐1 were purchased by ATCC (LGC Standards s.r.l., Sesto San Giovanni, Italy) and cultured in RPMI Medium 1640 (Thermo Fisher Scientific, Waltham, MA, USA) supplemented with 10% heat‐inactivated fetal bovine serum (FBS, Thermo Fisher Scientific), 1% penicillin and streptomycin (PS, Thermo Fisher Scientific), and 1% l‐glutamine (Thermo Fisher Scientific). THP‐1 were differentiated into M0 macrophages with 100 ng/mL phorbol‐12‐myristate‐13‐acetate (PMA, Sigma‐Aldrich, St. Louis, MI, USA) for 36 h followed by a resting period of 24 h; while M1 or M2 polarization was triggered for 24 more hours with 100 ng/mL lipopolysaccharide (LPS, Sigma‐Aldrich) plus 20 ng/mL IFN‐γ (Sigma Aldrich), and with 20 ng/mL IL‐4 (Sigma‐Aldrich), respectively. Conditioned media (CM) from THP‐1 polarized cells cultured on different surfaces were collected, centrifuged, and stored at −80°C until further use.

Human osteoblast‐like cells MG‐63 (ATCC) were cultured with high glucose Dulbecco Modified Eagle's Medium (DMEM, Biowest, Nuaillé, France) supplemented with 10% FBS, 1% PS, and 1% l‐glutamine. Osteogenic differentiation was induced by supplementing the standard culturing medium with or without 50% THP‐1 CM and with the osteogenic stimuli β‐glycerophosphate (10 mM) and ascorbic acid (250 µM, both from Sigma‐Aldrich) for 21 days, with medium exchange every other day.

### 2.4 Immunofluorescence (IF)

Cells were fixed in 4% buffered formalin (PFA, Sigma‐Aldrich) for 10 min at room temperature (RT), rinsed twice in Phosphate Buffer Saline (PBS), and permeabilized with a 0.1% solution of Triton X‐100 (Sigma‐Aldrich) for 5 min at RT. After two washings in PBS, samples were blocked with 10% Bovine Serum Albumin (BSA, Sigma‐Aldrich) and 3% Normal Goat Serum (NGS, Sigma‐Aldrich) for 90 min at RT. Primary antibodies anti‐human CD68 (Novus Biologicals, Bristol, United Kingdom), anti‐human CD86 (abcam, Cambridge, United Kingdom), and anti‐human CD206 (abcam) were diluted in 10% BSA and applied for 90 min at RT. After 2 washing in PBS, secondary anti‐mouse AlexaFluor 488‐ and an anti‐rabbit AlexaFluor 555‐conjugated antibodies (Thermo Fisher Scientific) were applied for 90 min at RT to detect the primary antibodies. After 3 final washing in PBS, cell nuclei were counterstained with DAPI (Sigma Aldrich) for 5 min at RT. Samples were finally coverslip‐mounted with the Dako Glycergel Mounting Medium (DAKO, Glostrup, Denmark) and analyzed with a Confocal Laser Scanning Microscope (Stellaris 5, Leica, Wetzlar, Germany).

### 2.5 Scanning Electron Microscopy (SEM)

Cells were fixed with a 2.5% glutaraldehyde solution (Sigma‐Aldrich) in 0.1 M Na‐Cacodylate buffer (Sigma‐Aldrich) for 30 min at RT, followed by washing in Na‐Cacodylate buffer for 5 min. Afterwards, samples were dehydrated in ethanol at increasing concentration (25%, 50%, 70%, 90%, 95%, 99%), critical point dried with liquid carbon dioxide (BALTEC, Wallruff, Germany), and sputter coated with a nanometer‐thick layer of gold through an SCD 040 coating device (Balzer Union, Wallruff, Germany). Images were taken using a dual‐beam Zeiss Auriga Compact system equipped with a GEMINI Field‐Effect SEM column (Zeiss, Oberkochen, Germany). SEM analysis was assessed at 5 keV.

### 2.6 Surface Elemental Analysis (EDX)

Energy‐dispersive X‐ray spectroscopy (EDX) was microanalytically performed to assess the elemental composition and confirm the selective presence of zirconium (Zr) on the R and modR surfaces compared to the SLA and modSLA controls (Figure S3). The analysis was conducted using the Zeiss SEM system at an increased accelerating voltage of 20 kV. Spectra were collected from several representative regions of each experimental group to accurately monitor the Zr‐Lα emission lines relative to the titanium (Ti) substrate matrix.

### 2.7 RNA Extraction, cDNA Synthesis and Quantitative Real Time‐Polymerase Chain Reaction (qRT‐PCR)

Total RNA from THP‐1 cells (M0, M1, and M2) and MG‐63 osteoblasts cultured in the presence of conditioned medium obtained from THP‐1 cultures was extracted using the RNeasy Mini Kit protocol (Qiagen, Hilden, Germany) according to the manufacturer's protocol for eucaryotic cells, and quantified using a P330 nanophotometer (Implen, Thermo Fisher Scientific). Five‐hundred micrograms of RNA were used as a template for cDNA synthesis using the High‐Capacity cDNA Reverse Transcription Kit (Applied Biosystems, Foster City, CA, USA). qRT‐PCR analysis was performed using Taqman probe sets *IL‐6* (Hs00174131_m1), *IL‐8* (Hs00174103_m1), *TNF‐α* (Hs00174128_m1), *IL‐10* (Hs00961622_m1), *CD206* (Hs07288635_g1), *ARG2* (Hs00982833_m1), (Applied Biosystems, Thermo Fisher Scientific), ALP (Hs01029144_m1), OPN (Hs00950010_m1), OCN (Hs001587814_g1), RUNX2 (Hs00231692_m1) and Col1α1 (Hs00164004 _m1) with a StepOne Plus Real‐Time PCR System (Applied Biosystems, Thermo Fisher Scientific).

Data were analyzed using the ΔΔCt method after normalization to the housekeeping gene GAPDH (Hs02758991_g1).

### 2.8 Immunoassay

IL‐4, ‐6, ‐10, and ‐17, Bone Morphogenetic Protein‐2 (BMP2), Osteoprotegerin (OPG), Osteopontin (OPN), TNF‐α, and Vascular Endothelial Growth Factor A (VEGF‐A) concentrations in THP‐1 CM were measured using the Luminex MagPix (Bio Rad Laboratories, Hercules, CA, USA), following the manufacturer's recommendations. In addition, macrophage polarization was assessed by quantifying the number of cells differentiated into M1 or M2 phenotypes. Quantification was performed by image‐based cell analysis using NIS‐Elements BR software (version 5.11; Nikon, Tokyo, Japan).

### 2.9 Alizarin Red Staining

Culture mineralization was assessed using Alizarin Red Staining (Sigma‐Aldrich). Briefly, cells were washed twice with PBS and fixed with 4% PFA for 15 min at RT, then rinsed twice with double‐distilled water (ddH_2_O) and incubated with a 40 mM Alizarin Red S solution (pH 4.2) for 40 min at 4°C. After thorough washing with ddH_2_O, samples were air‐dried before imaging with a stereomicroscope (SMZ25, Nikon). Following image acquisition, the concentration of Alizarin Red S was measured by solubilizing the dye with 10% acetic acid (Sigma‐Aldrich) and reading the absorbance at 405 nm on a Multiskan FC microplate reader (Thermo Fisher Scientific) using a standard reference curve.

### 2.10 Statistical Analysis

Data were analyzed using Prism 8 (GraphPad, La Jolla, CA, USA). All the values are reported as the mean ± standard deviation (SD) of three independent experiments with six biological replicates. Differences between groups were evaluated with the one‐way and two‐way analysis of variance (ANOVA) with Tukey's post hoc test, after having assessed the normality of the data using the Shapiro–Wilk normalization test. Differences were considered significant when *p* < 0.05.

## 3 Results

### 3.1 Addition of Zirconia to Titanium Implant Surface Promotes the Pro‐Inflammatory M1 Polarization

**Figure 1:**
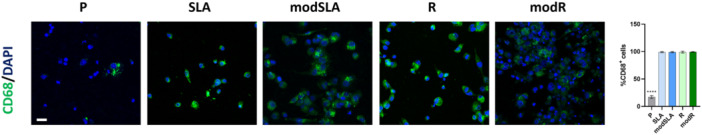
Monocyte‐to‐macrophage transition on dental implant‐like surfaces. Immunofluorescence images of THP‐1 cells polarized into steady‐state M0 macrophages on different implantable surfaces. The histogram reports the quantification of CD68‐positive cells found on each surface. ****p < 0.0001. Scale bar: 25 μm.

Immediately after implant placement, circulating blood monocytes face the implantable surface and differentiate into steady‐state (M0) macrophages. To reproduce this scenario in vitro, we cultured THP‐1 cells on P, SLA, modSLA, R, and modR surfaces and treated them with PMA and stained for CD68 positivity, to confirm the presence of M0 phenotype (Figure 1).

The expression of the steady‐state M0 differentiation marker CD68, as well as the quantification of the positive cells, reveals a better activation of the macrophages on titanium surfaces with a microrough topography (SLA, modSLA, R, modR) compared to the smooth (P) counterpart. These data are in line with previous results (Parisi et al. 2020) and confirm the suitability of our model to study macrophage‐implant surface interaction.

**Figure 2:**
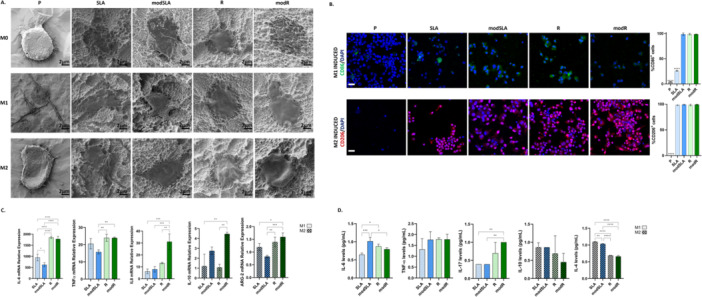
Macrophage activation on dental implant‐like surfaces. (A) SEM images of THP‐1 cells induced into steady‐state macrophages (M0) or polarized into M1 and M2 macrophages on different implantable surfaces. Scale bar: 2 µm. (B) Immunofluorescence images of CD86 (green) and CD206 (red) positive cells on different implantable surfaces. Histograms to the right report the quantification of CD86‐ and CD206‐positive cells found on each surface. ****=*p* < 0.0001. Scale bar 25 μm. (C) mRNA levels of IL‐6, TNF‐α, and IL‐8, and of IL‐10, and ARG‐2 on different implantable surfaces, after M1 or M2 polarization, respectively. ****p < 0.0001, ***p = 0.0008, **p = 0.0051, and *p = 0.0365. (D) Quantification of secreted pro‐inflammatory (IL‐6, TNF‐α, and IL‐17) and anti‐inflammatory (IL‐4 and IL‐10) cytokines on different implantable surfaces, after M1 and M2 polarization, respectively. ****p < 0.0001, ***p = 0.0009, **p = 0.0080, and *p = 0.0209.

Next, we wanted to investigate whether the different chemical composition (SLA or R) and the hydrophilicity (modSLA or modR) could further influence or even revert the induced M1/M2 polarization of the macrophages (Figure 2). While the morphology of the cells was similar on all the considered surfaces (Figure 2A), the CD86 expression (Figure 2B) revealed that the M1 phenotype was promoted by the presence of zirconia (R and modR) and of titanium hydrophilicity (modSLA). Notably, on the P surface, which failed in supporting the monocyte‐to‐macrophage transition, M1 or M2 differentiation also could not be achieved, confirming the idea that roughness plays a key role in macrophage functioning. Our results were partly confirmed by Real Time‐PCR (Figure 2C) and Luminex analysis (Figure 2D).

Indeed, the presence of zirconia (R and modR) promoted a higher transcription and secretion of pro‐inflammatory cytokines, with statistically significant increases observed for IL‐6 and IL‐17 (Figure 2D), while a similar upward trend was found for TNF‐α and IL‐8. Consistently, although we observed a similar number of CD206‐positive cells on all the tested surfaces (with the exception of P), while investigating the cytokine expression profile, we observed that the presence of zirconia significantly inhibited the secretion of IL‐4, while a non‐significant reduction was observed for IL‐10 (Figure 2D).

Taken together, all these results suggest that the surface characteristics in terms of chemical composition of the implantable surface play a key role in determining the early macrophagic phenotype transition. In contrast, the hydrophilicity of the surface, even if different among the groups (Figure S1) does not seem to be relevant to the early M1/M2 activation.

### 3.2 M1 Polarization Is Pivotal for the Creation of a Pro‐Osteogenic Environment and the Presence of Zirconia in the Implantable Surface Boosts the Release of Cytokines Favorable for Bone Formation

**Figure 3:**
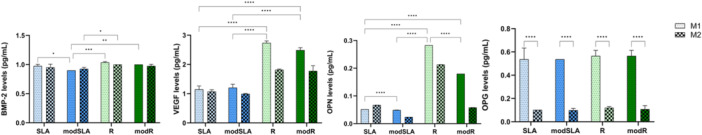
THP‐1 secretion of bone‐stimulating factors. Quantification of secreted BMP2, OPG, OPN, and VEGF‐A on different implantable surfaces, after M1 and M2 polarization, respectively. ****p < 0.0001, ***p = 0.0002, **p = 0.0050, and *p = 0.0487.

Next, it looks fundamental to understand if the activation of macrophages was correlated with the secretion of cytokines associated with new bone formation. Hence, we quantified the amount of bone morphogenetic protein 2 (BMP‐2), vascular endothelial growth factor (VEGF‐A), osteopontin (OPN), and osteoprotegerin (OPG) in the supernatant of THP‐1 macrophages polarized as either M1 or M2 on SLA, modSLA, R, and modR surfaces (Figure 3). The levels of BMP‐2 were surprisingly higher in the M1 groups of R, modR, and SLA samples, if compared to modSLA. Again, the presence of zirconia resulted in an increase in the secretion of BMP‐2 in R if compared to modSLA. Similarly, the type of surface also did not influence the amount of OPG release, but the M1 activation promoted a stronger release of OPG compared to the M2 activation on all the tested surfaces, suggesting that an initial M1 polarization is essential to trigger the release of cytokine capable of activating osteoblasts. In line with this, stronger secretion of VEGF and OPN was also observed for M1 polarization, secretions that were further promoted by the presence of zirconia. Taken together, our data suggest that M1 activation is a fundamental step to allow the formation of an environment favorable to drive new osteogenesis, and that the implantable surface has a role in controlling the release of specific cytokines.

### 3.3 The Presence of Zirconia in the Implantable Surface Contributes to the Mineralization of Human Osteoblasts, Similarly to an Enhanced Hydrophilicity of a Titanium Surface

We observed that the polarization of macrophages into the M1 phenotype seems to be essential for the release of cytokines supporting new bone formation.

**Figure 4:**
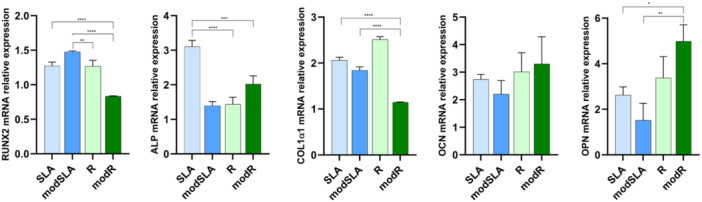
Osteogenic gene expression profile of MG‐63 cells after 21 days of culture with conditioned media (CM) derived from THP‐1 M1 macrophages cultured on SLA, modSLA, R, and modR surfaces. Relative mRNA expression levels of RUNX2, ALP, COL1α1, OCN, and OPN in MG‐63. *p = 0.02, **p < 0.05, ***p = 0.005, and ****p < 0.0001.

This effect was further corroborated at the molecular level by the mRNA expression patterns of key osteogenic markers at day 21 (Figure 4), which reflected a coordinated progression toward mature osteogenesis. This advanced differentiation stage was evidenced by the marked downregulation of the early marker ALP in both R and modR conditions compared to SLA, accompanied by a reduction of RUNX2 and the early matrix gene COL1α1 specifically in the modR group. Conversely, while the expression of the late marker OCN remained unaffected without significant differences among the various surfaces, OPN expression was markedly upregulated in the zirconia groups, reaching its peak in the modR environment. Collectively, these molecular findings confirm the transition of MG‐63 cells toward a mature osteoblastic phenotype under the paracrine influence of zirconia‐activated macrophages.

**Figure 5:**
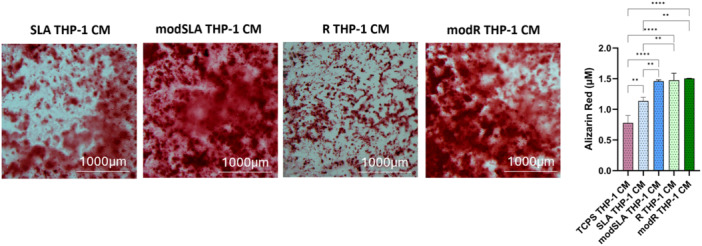
MG‐63 mineralization after THP‐1‐to‐M1 CM exposition obtained by culture on different implantable surfaces. Alizarin Red staining of MG‐63 cells (red) 21 days after osteogenic induction in the presence of CM derived from THP‐1 cells differentiated into M1 macrophages on SLA, modSLA, R, and modR discs. The histogram to the right reports the Alizarin Red quantification. Scale bar: 1000 μm; **p = 0.0018 TCPS vs. SLA, *p* = 0.0039 SLA vs. modSLA, *p* = 0.0026 SLA vs. R, and *p* = 0.0014 SLA vs. modR; ****p < 0.0001.

Therefore, we lastly exposed human osteoblast‐like cells MG‐63, pre‐cultured on standard tissue culture polystyrene (TCPS), to the collected CM of THP‐1 polarized into M1 macrophages on the different implantable surfaces, and assessed their capacity to mineralize after 21 days (Figure 5). By culturing MG‐63 cells exclusively on TCPS, we isolated the biochemical effects of the macrophage‐released soluble factors from any direct topographical influence of the implant surfaces, which has been assessed by Alizarin Red staining and Real Time‐PCR analysis. The CM derived from THP‐1 M1 cultured on the R and the modR surfaces promoted the mineralization of MG‐63 cells compared to the SLA, without significant differences between the two surfaces. Interestingly, the hydrophilicity of the SLA surface (modSLA) also promoted mineralization to a similar extent as the R and modR surfaces. Taken together, these results suggest that the addition of zirconia to the implantable surface boosts the mineralization of osteoblastic cells as much as hydrophilicity, confirming that it seems to be the presence of zirconia that favors the creation of an immune environment favorable to support osteoblastic mineralization.

## 4 Discussion and Conclusion

After dental implant positioning, circulating monocytes are the first cells that approach the surface, rapidly differentiating into macrophages that play an essential role in controlling the healing process and osseointegration.

Over the past decade, this phenomenon has been increasingly reinterpreted as an osteoimmune process rather than a purely bone‐centric healing process. Albrektsson et al. (2023) formally proposed that endosseous implants should no longer be considered as bioinert but rather as osteoimmunomodulatory materials that actively shape the immune microenvironment and influence downstream regenerative events; in particular, osseointegration was described as a dynamic foreign body equilibrium regulated by macrophage behavior and its balance over time. More recently, Yun et al. (2025) expanded this concept, including additional immune regulators of the peri‐implant immune environments, such as T‐regulatory cells, further underscoring the complexity of the immune‐bone axis.

Against this background, the objective of the present study was to investigate how implant surface characteristics influence macrophage polarization and how the resulting macrophage‐derived microenvironment affects osteoblasts activation and bone formation. Macrophages phagocyte and neutralize bone debris generated during insertion and subsequently, secrete cytokines to recruit bone‐forming cells to the implant surface. Clinically, the timing of these events is known to depend on implant surface characteristics, including microtopography, wettability, and chemical composition; however, a full mechanistic understanding of how these parameters influence immune cell activation and, consequently, bone‐forming cell behavior, is still lacking (Hotchkiss et al. 2016; Alfarsi et al. 2014).

The present study aligns with this paradigm by demonstrating that zirconia‐enriched titanium surfaces directly modulate macrophage phenotype and, through paracrine signals, enhance osteoblast mineralization. These findings reinforce the concept that the immune microenvironment established at the implant interface is a decisive determinant of bone formation outcomes.

Our data show that monocyte‐to‐macrophage (M0) differentiation, as evidenced by CD68 expression, was significantly higher on microrough surfaces (SLA, modSLA, R, modR) than on the polished control (P), confirming the well‐established role of surface topography in promoting macrophage adhesion and activation. In addition, previous studies specifically focused on SLA and modSLA titanium have shown that microrough surfaces support macrophage engagement and polarization, while hydrophilic modifications can modulate cytokine profile towards a less inflammatory phenotype (Shirazi et al. 2022; Donohoe et al. 2023). Our results partially aligned with this picture: surface roughness was necessary for macrophage activation in our model, whereas hydrophilicity, intrinsically linked to the underlying surface chemistry, did not exert the dominant immunomodulatory effect attributed to it in studies on pure titanium surfaces (Alfarsi et al. 2014; Hotchkiss et al. 2016). This discrepancy may be explained by the additional contribution of zirconia chemistry, which appears to introduce a competing and potentially stronger signal capable of overriding the wettability‐driven effect on macrophage behavior.

It has been previously established that surface hydrophilicity is pivotal in promoting clinical osseointegration, as evidenced by the superior performance of modified hydrophilic surfaces (e.g., modSLA) over less hydrophilic alternatives (e.g., SLA) (Hao et al. 2021; Abdel‐Haq et al. 2011). Complementary in vitro studies support these observations, showing that hydrophilicity improves osteoblast differentiation and mineralization (Parisi et al. 2020; Toffoli et al. 2020). While our findings do not contradict these data, they suggest that chemical composition, and specifically the presence of zirconia, may represent an equally potent and mechanistically distinct driver of the osteoimmunomodulatory response.

A central finding of this study is that zirconia‐containing surfaces (R and modR) promoted an early M1‐like macrophage polarization, as evidenced by elevated CD86 expression and increased transcription and secretion of IL‐6, TNF‐α, IL‐8, and IL‐17, accompanied by suppression of IL‐10 and IL‐4. These results partially diverged from prior reports on hydrophilic titanium/zirconia surfaces: a systematic review published in 2022 reported that the hydrophilic Roxolid SLActive alloy (the closest in terms of chemical composition to our modR surface) tended to downregulate pro‐inflammatory cytokines and induce an M2‐like anti‐inflammatory microenvironment when compared to hydrophobic SLA. However, those conclusions were drawn predominantly from studies using murine macrophages, whereas our study employed the THP‐1 human monocyte‐derived model, which may respond differently to surface chemistry (Pitchai et al. 2022). Moreover, prior work did not systematically distinguish between the effect of zirconia chemistry per se and the effect of hydrophilicity, whereas our experimental design, comparing SLA versus mod SLA and R versus modR in parallel, allowed these two variables to be partially decoupled, revealing surface composition as the dominant immunomodulatory signal. In our dataset, the modR surface, which includes both features, did not show clear advantages over R alone, suggesting that added hydrophilicity may not enhance the response of zirconia‐containing surfaces. In contrast, increased hydrophilicity improved osteoblasts mineralization in SLA‐type surfaces. Future studies employing a factorial design, in which zirconia content and hydrophilicity are varied independently across surface‐matched substrates, would be needed to formally quantify the interaction between these two parameters.

Importantly, M1 polarization in our model was not associated with a purely pro‐inflammatory outcome; rather, it was coupled with increased secretion of pro‐osteogenic mediators (i.e., OPG, BMP‐2, VEGF, and OPN) on all the tested surfaces. This repositions M1 macrophages as active contributors to the osteogenic niche rather than merely inflammatory effectors. In particular, zirconia‐containing surfaces further amplified the release of VEGF‐A and OPN compared to SLA‐like surfaces. VEGF‐A couples angiogenesis with osteogenesis by stimulating osteoblast differentiation and facilitating vascular invasion of the newly forming bone matrix, while OPN serves as a bridging molecule that anchors osteoblasts to the mineralized matrix and potentiates M1‐driven bone remodeling signals (Liu et al. 2017; Hu and Olsen 2016a, 2016b). Together, these data suggest that zirconia‐containing surfaces may more effectively support the osteogenic microenvironment than SLA‐like counterparts. Consistently, titanium/zirconia implants have demonstrated greater stability compared to pure titanium ones, likely reflecting both mechanical and biological advantages (Rohr et al. 2020).

Although zirconia‐loaded surfaces induced an early M1‐like macrophage response, this should not be interpreted as a purely detrimental effect. A transient pro‐inflammatory phase is considered necessary for debris clearance and for initiating the sequence of events that leads to tissue repair and osseointegration. Macrophage polarization is a dynamic rather than binary process, and early M1 activation can coexist with the release of pro‐osteogenic mediators that support angiogenesis and bone formation, including BMP‐2, OPN, and VEGF‐A (Zhang et al. 2023; Shi et al. 2025). In this context, the apparent “dual” behavior observed on zirconia‐containing surfaces may reflect a productive osteoimmune response in which early immune activation promotes the local signaling environment required for subsequent regeneration, rather than presenting a simple inflammatory disadvantage.

The beneficial role of zirconia may therefore lie not in suppressing macrophage activation, but in favor of an early and functionally coordinated immune response that supports bone remodeling and osseointegration (Rohr et al. 2020).

To further confirm the pro‐osteogenic effect of the M1‐conditioned microenvironment, a quantitative mRNA expression analysis of key osteogenic markers in MG‐63 cells was performed after 21 days, in order to maintain the same experimental time points of the calcium nodules production as a consequence of the mineralization process. The apparent downregulation of ALP and RUNX2 in R and modR groups is not a sign of impaired differentiation, as both are early markers known to decline as osteoblasts reach terminal maturation. Indeed, ALP expression peaks in the first days of osteogenic induction and rapidly declines as cells progress toward a mineralizing phenotype, with RUNX2 similarly showing a trend of increasing and then decreasing with osteoblast maturation (Zhou et al. 2021; Li et al. 2022). Conversely, the significant upregulation of OPN in R and modR groups marks late‐osteogenic commitment, consistent with recognized role as a late differentiation marker alongside OCN (Li et al. 2020). These transcriptional patterns are further supported by recent implant surface studies showing that surface‐driven immunomodulation directly shapes the osteogenic transcriptional program, with enhanced OPN, ALP, and OCN expression observed at 14 and 21 days in osteoblasts exposed to macrophage‐conditioned medium from nanoporous titanium surfaces, and zirconia coatings significantly increase osteoblast‐specific gene expression in hMSCs compared to acid‐etched controls (Moon et al. 2024; Mendonça et al. 2009). Collectively, these findings corroborate the Alizarin Red mineralization data and support the conclusion that zirconia‐based surfaces promote a more advanced osteogenic program through M1 macrophage paracrine signaling, in line with the broader principle that macrophage‐derived osteconductive factors upregulate markers including RUNX2, ALP, OCN, and OPN.

Although the results of the present study are interesting and contribute to expanding the understanding of the molecular basis of osseointegration, they are not free from limitations. We must acknowledge that our model is a simplified model and, while allowing mechanistic dissection of the immune‐osteogenic axis, lacks the spatial and temporal complexity of the peri‐implant healing in vivo, including vascular infiltration, osteoclast activity, and mechanical loading. Furthermore, the use of the THP‐1 and MG‐63 cell lines, although widely adopted for mechanistic studies and screening, may not fully reflect the biology of healthy primary cells, limiting the fidelity with which the in vivo transcriptomic and metabolomic environment is recapitulated. To address these limitations, future research should explore more sophisticated models using macrophages derived from circulating human healthy monocytes (Parisi et al. 2023) and primary bone cells, and incorporate extended time‐course analysis to better elucidate the dynamics of M1‐to‐M2 transition, an increasingly recognized variable in determining tissue repair outcomes (Parisi et al. 2023).

In conclusion, this study demonstrates that the addition of zirconia to the titanium formulation promotes M1 macrophage activation, boosts the release of pro‐osteogenic cytokines, including VEGF‐A and OPN, and improves indirect osteoblasts mineralization to a level comparable to enhanced hydrophilicity. These effects appear to be primarily driven by surface chemical composition rather than wettability alone. Even if a consensus on the exact role of macrophage polarization at the peri‐implant site has not yet been reached, the M1/M2 balance clearly plays a key role in determining whether tissue disruption or regeneration prevails (Li et al. 2024).

Overall, our findings support the interpretation of osseointegration as an osteoimmune process rather than a classic bone healing response, reinforce the importance of early macrophage activation in establishing a favorable osteogenic microenvironment, and suggest that the dysregulation of M1/M2 balance may underlie peri‐implant bone loss (Albrektsson et al. 2023). Finally, the use of titanium/zirconia alloys may provide an osteoiummunomodulatory advantage that complements their established mechanical benefits, warranting further preclinical and clinical investigations (Albrektsson et al. 2023).

## Author Contributions

Conceptualization: Benedetta Ghezzi, Ludovica Parisi, and Simone Lumetti. Methodology: Ottavia Cannatella, Benedetta Ghezzi, and Ludovica Parisi. Validation: Ottavia Cannatella, Benedetta Ghezzi, and Ludovica Parisi. Formal analysis: Ottavia Cannatella, Biagio Matera, and Francesca Rossi. Investigation: Ottavia Cannatella, Benedetta Ghezzi, Francesca Rossi, and Ludovica Parisi. Resources: Benedetta Ghezzi, Ludovica Parisi, and Simone Lumetti. Data curation: Ottavia Cannatella, Biagio Matera, and Giovanni Passeri. Writing – original draft preparation: Ottavia Cannatella and Biagio Matera. Writing – review and editing: Benedetta Ghezzi, Ludovica Parisi, and Giovanni Passeri. Visualization: Benedetta Ghezzi, and Ludovica Parisi. Supervision: Benedetta Ghezzi and Giovanni Passeri. Project administration: Benedetta Ghezzi. Funding acquisition: Benedetta Ghezzi, Ludovica Parisi, and Simone Lumetti. All authors have read and agreed to the published version of the manuscript.

## Ethics Statement

This study was conducted entirely in vitro, using cell cultures and synthetic models. No human or animal subjects were involved in the research. All procedures were performed in accordance with relevant institutional guidelines for laboratory‐based research. The materials and methods used in this study were chosen with the utmost care to ensure scientific validity and safety, without any impact on living organisms. Ethical approval was not required for this study due to the absence of human or animal involvement.

## Conflicts of Interest

The authors declare no conflicts of interest.

## Supporting information


Supporting File

## Data Availability

The data that support the findings of this study are available from the corresponding author upon reasonable request.
