# Supplementary material for: Osteoimmunomodulatory Effects of Zirconia‐Modified Titanium: Promoting Macrophage Activation and Osteoblast Mineralization at the Dental Implant Interface
Source: Clin Exp Dent Res. 2026 Jul 19;12(4):e70414. doi: 10.1002/cre2.70414 (PMC13380818; doi:10.1002/cre2.70414)
Supplement: Supplementary file 1 — Supporting File [file CRE2-12-e70414-s001.docx]

**SUPPLEMENTARY MATERIALS**

**Title: Osteoimmunomodulatory effects of zirconia-modified titanium: promoting macrophage activation and osteoblast mineralization at the dental implant interface**

**Running title: Osteoimmunomodulatory effects of zirconia-titanium surfaces**

Ottavia Cannatella^1,2$^, Biagio Matera^1,2$^, Francesca Rossi^3^, Giovanni Passeri^2^, Simone Lumetti^1,2,3^, Ludovica Parisi^4#^, Benedetta Ghezzi^1,2#^

^1^Center of Dental Medicine, University of Parma, Via Gramsci 14/A, 43126 Parma, Italy.

^2^ Department of Medicine and Surgery, University of Parma, Via Gramsci 14/A, 43126 Parma, Italy.

^3^ IMEM-CNR, Institute of Materials for Electronics and Magnetism-National Research Council, Parco Area delle Scienze 37/A, 43124 Parma, Italy.

^4^Laboratory for Oral Molecular Biology, Department of Orthodontics and Dentofacial Orthopedics, University of Bern, Bern, Switzerland.

^$^Equal contribution of the authors

^#^Equally contribution of the authors

**Surface hydrophilicity evaluation**

SLA (θ = 110.34° at T0 and θ = 108.95° at T30) and R (θ = 120.75° at T0 and θ = 118.19° at T30) surfaces were classified as hydrophobic, while modSLA (θ = 15.21° at T0 and θ = 7.47° at T30) and modR (θ = 13.55° at T0 and θ = 10.98° at T30) surfaces were classified as super-hydrophilic. **Figure S1** shows water droplets on discs of the four materials during contact angle measurement.


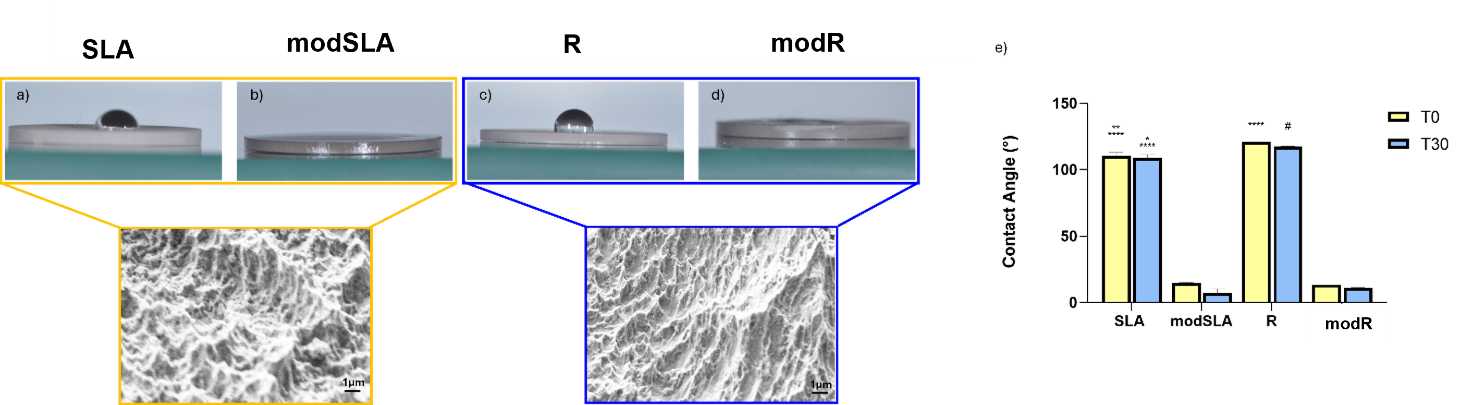


Figure S1: Water contact angle measurements on titanium discs: a) SLA, b) modSLA, c) R and d) modR. e) T0 and T30 correspond to the θ measured when the drop encounters the material surface. **** p<0.0001 SLA vs. modSLA, SLA vs. R+, modSLA vs. R; # p<0.0001 R vs. modR; ** p=0.0041 SLA vs. R.

**Surface roughness analysis by profilometry**


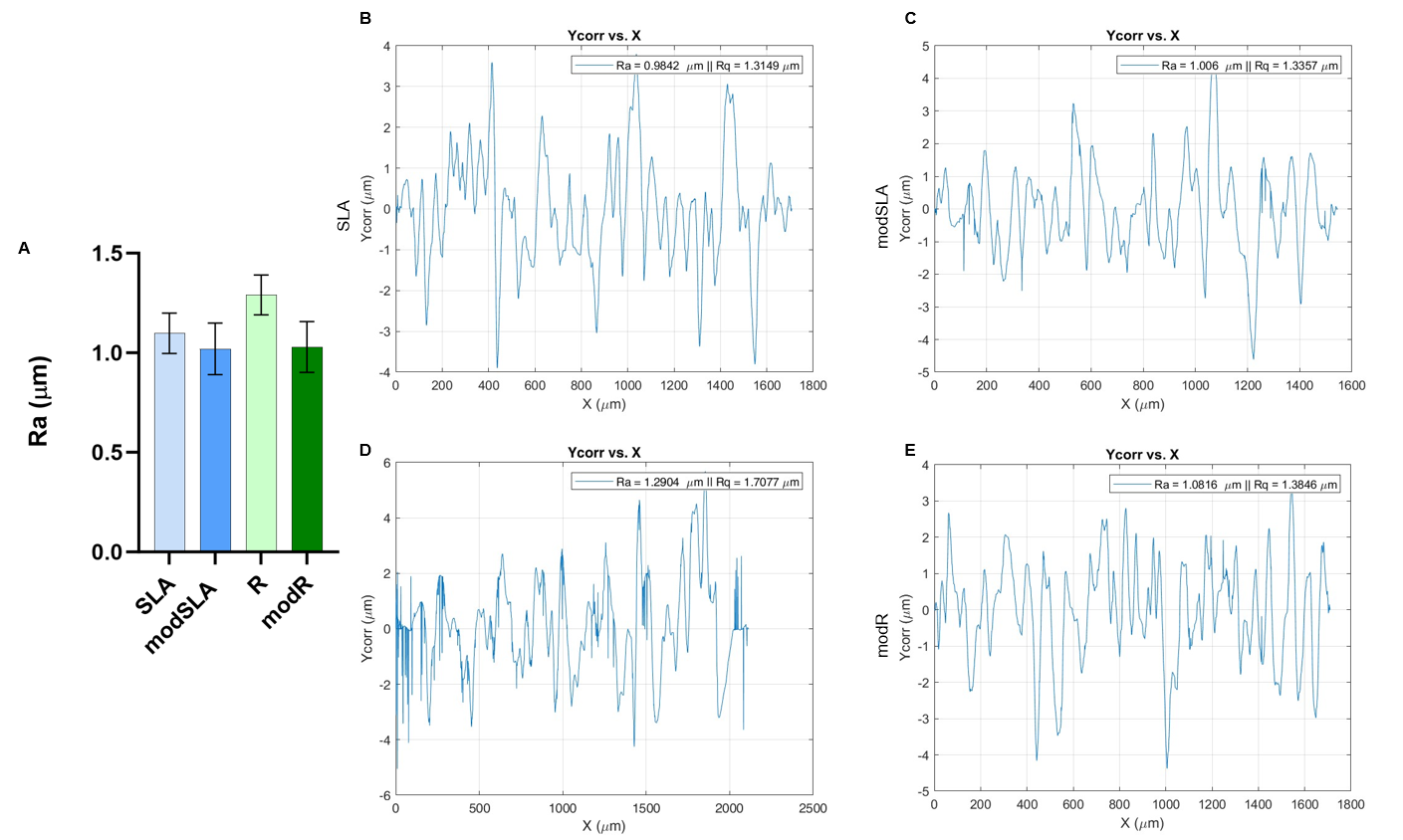


Figure S2: Surface micro-topography. Profilometric analysis of titanium surfaces roughness a) Mean roughness values (Ra) obtained from profilometer measurements for SLA, modSLA, R, and modR surfaces. b-e): Representative profilometric traces (Ycorr vs. X) for each surface: b) SLA; c) modSLA; d) R; and e) modR.

**Surface Elemental Characterization (EDX)**

To address the evaluation of the surface elemental composition and verify the zirconium (Zr) incorporation, EDX microanalysis was performed on all experimental groups (Figure S3).


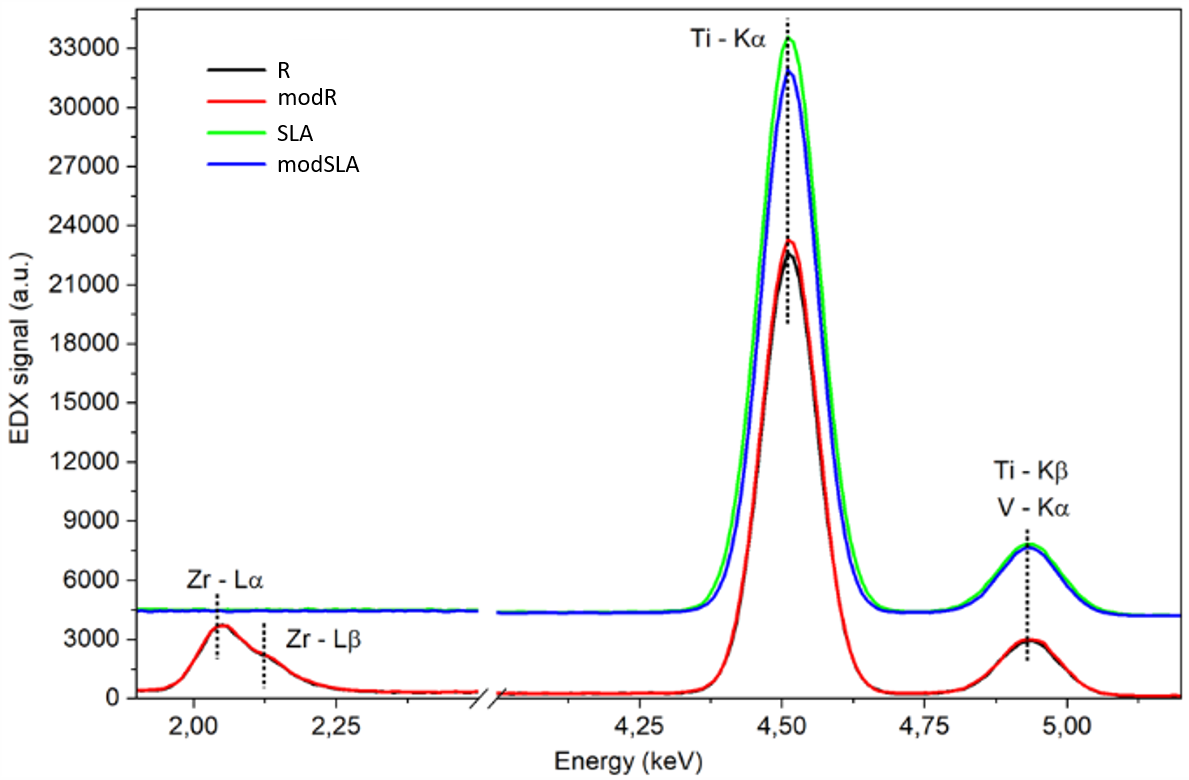


Figure 3S: EDX spectra of the experimental surfaces (R, modR, SLA, and modSLA).

The acquired spectra for the SLA and modSLA groups showed no detectable signal for zirconium, with the profiles remaining entirely flat around the 2.04 keV region; these surfaces were dominated exclusively by the characteristic peaks of the titanium substrate (Ti-Kα at ~4.51 keV and the overlapped Ti-Kβ / V-Kα at ~4.93 keV). In contrast, the spectra for both the R and modR groups clearly revealed the successful incorporation of zirconium, as evidenced by the distinct Zr-Lα emission peak at ~2.04 keV and the accompanying Zr-Lβ peak. Notably, the EDX curves for R (black line) and modR (red line) are perfectly overlapped in the zirconium energy region, indicating a highly consistent and comparable presence of the Zr element between these two specific surfaces. These qualitative findings successfully confirm the selective presence of zirconium restricted to the R-series materials.
